# Supplementary material for: Anatomy education potential of the first digital twin of a Korean cadaver
Source: PLoS One. 2025 Mar 10;20(3):e0305679. doi: 10.1371/journal.pone.0305679 (PMC11893122; doi:10.1371/journal.pone.0305679)
Supplement: Attachment — Submitted filename: Reviewer_comments_PlosOne_20240805_v1.docx [file pone.0305679.s004.docx]

**Reviewer Comments:**

**Reviewer #1:**

1. introduction: informative but the main aim of the research should be briefly addressed at its end

Thank you for your comment.

The main purpose of the study has been added at the end of the introduction.

L116:

In essence, this research aims to pioneer XR anatomy education developing a 3D DT of a KSMC in the metaverse, introducing transformative and collaborative approaches to current challenges and future possibilities in medical education and telemedicine.

2. methodology: presented with detailed structure

Thank you for your comment regarding the detailed structure of the methodology section. We have made further enhancements to the clarity and comprehensiveness of the "Materials and Methods" section to ensure it accurately conveys the process and techniques used in developing the 3D Digital Twin Cadaver and the accompanying XR content for the metaverse platform.

Before revision of the version methodology structure is as follows.

**MATERIALS AND METHODS**

**From Cadaver to a 3D Digital Twin Cadaver**

Collecting and Scanning

Refining and Retopologizing the Mesh for Accuracy

Optimizing Polygons

**Architecture for the metaverse platform to Facilitate a 3D digital twin**

HoloLens 2

Developing a Metaverse Platform and Adding Interactivity

**Development of XR contents**

The revised methodology structure is as follows:

**MATERIALS AND METHODS**

**From Cadaver to a 3D Digital Twin Cadaver**

Collecting and Scanning

Refining the Mesh

Retopologizing the mesh

Optimizing Polygons

**The architecture to facilitate the 3D DT in metaverse**

HoloLens 2

Developing a Metaverse Platform

Developing real-time interaction features between users

Enhancing XR content with remote rendering

**Pilot test feedback**

3. results: great effort is recognized addressing short video for the actual usage of the system would have been a marked

We sincerely appreciate your suggestion and believe that a video can be an effective supplement to our Manuscript.

L414:

A short video showcases few moments from pilot tests and provides a walkthrough of the developed Metaverse platform and the 3D DT of the KSMC, called ‘Skeletomy.’ This video, included as supplementary material, provides an immersive experience from various perspectives. Two users are featured in the video: the main user (female) is shown from a 1st person perspective, and the secondary user (male) is shown in 3rd person perspective. Together, they demonstrate real-time collaboration features as they interact with the same 3D digital twin (DT) of the Korean Standard Male Cadaver (KSMC). Through this video, viewers also get a chance to immerse themselves in an indirect experience of learning with the KSMC and to envision a new virtual era for applications in anatomy education and telemedicine.

4. Result.- addressing any reviews of the users either students or professors would have been a plus OR it may be a complementary research work???!!!

Per your suggestion, we added some quotes and comments from anonymous reviews and feedback that we gathered from students, professors, and professionals in the medical field. We agree that adding these would enhance the solidity of this article.

Consequently, we have included several anonymous reviews and feedback—not only highlighting honest pros and cons but also offering perspectives on potential improvements for complementary research.

L246:

**Pilot test feedback**

The Metaverse platform and the 3D DT content developed in our study underwent a thorough process of gathering pilot test feedback from anonymous participants in the medical field, including company employees, students and professors from medical and nursing schools. This feedback was collected through an open-ended survey covering general inquiries and interests, evaluations of quality and potential adoptability, and suggestions for further improvements. No participants were required to answer all categories, resulting in some respondents addressing one, two, or all of the topics.

L317:

**Pilot test feedback**

**Group of anonymous college professors**

College professors expressed interest in the applicability of the Metaverse platform across different departments. Majority of their inquiries focused on how the platform could be integrated into school curricula and whether additional content developments would occur to accommodate specific medical departments like ophthalmology, dentistry, cardiology or even veterinary medicine.

**Group of anonymous students**

A participant in an open-ended survey among nursing students who engaged with our program remarked, “Memorizing bone structures from a single diagram has always been a challenge for me. That’s why my friends and I always draw diagrams and color them to memorize them for exams. This metaverse program, however, is very fun interacting with each part of the bone. It’s like floating bones with quiz cards.” Numerous students expressed appreciation for the interactive elements and the capability for real-time collaboration. Another student noted, “When it comes to learning anatomy, I think it’s crucial for us to quiz each other and make the learning process more efficient. However, during the COVID-19 restrictions, it was extremely difficult for us to study because none of us were able to study in groups. If we had been provided with this program, I believe it would have been much easier and more enjoyable to have a group of our classmates join a session together to interact with the same cadaver in real-time.”

**Group of anonymous corporate guests**

Although the research and pilot testing were not specific intended for buyers, many corporate guests expressed interest in the pricing of available content on the platform and the overall timeline for the development of the upcoming contents. Meanwhile, some corporate guests were particularly fascinated by the originality of the content and wanted to know if major companies or organizations were already using it. Some reported frequent lags between movements in the scene, which may be due to the quality of internet speed depending on the environment’s connection. This highlights the need for optimize the connection between the platform and the internet to ensure a seamless user experience, regardless of internet speed and stability.

**Feedback from the demo session at a medicine conference**

Feedback from the demo session at an medicine conference highlights the ultimate benefits and necessity of the platform in fields that require an accurate understanding of human anatomy, such as Oriental medicine, particularly acupuncture. Users found that viewing the human skeleton in 3D improved comprehension and teaching performances. They expressed fascination with the interactive features that allowed them to zoom in, zoom out, and rotate the skeletal model. All participants at the demo session were optimistic about this research, envisioning potential collaboration between Western and Asian medicine to form a creative, integrated solution in medical field.

AR elements have been evaluated as effective in improving learners' concentration and immersion. This is a huge advantage in educational settings, promoting better engagement and understanding.

The platform's potential to replace traditional hands-on methods using live cadaver dissections or physical models was highly praised for its efficiency. Users highlighted benefits such as cost savings, solving hygiene issues and easing constraints in the physical practice environment.

5. discussion: though it is clear that it is a denovo system but i would have prefered to compare it to other 3d systems that may be used in teaching anatomy, or address its advantages to well known 2d systems and so on

We are grateful for the insightful feedback provided by the reviewer. Your comment highlighted the importance of contextualizing our de novo 3D system within the broader landscape of anatomical teaching tools. In response, we have added a comparative analysis to the discussion section of our manuscript.

Specifically, we have included a comparison of our 3D system with other 3D models, such as physical 3D printed models used in cardiology and otolaryngology training, and with VR and AR technologies. This addition emphasizes the tactile advantages and immersive experiences offered by these systems (25, 26, 27).

Additionally, we addressed the advantages of our 3D system compared to traditional 2D systems, highlighting its ability to provide a more comprehensive and interactive understanding of anatomical structures, which is often lacking in 2D representations.

L359:

In the development and application of our novel de novo 3D system for anatomical education, it is important to compare its efficacy and advantages against other established 3D and 2D systems used in teaching anatomy. Although our system presents unique features, understanding its relative performance is crucial for assessing its potential impact.

Physical 3D printed models, such as those utilized in cardiology training, offer valuable feedback and interactive experiences that enhance the learning process. Studies have demonstrated that these models can significantly aid in developing a practical understanding of complex anatomical structures (25). For example, physical models allow learners to engage in hands-on manipulation, which is instrumental in reinforcing spatial relationships and anatomical details.

In the field of otolaryngology, 3D printed models created from cadaveric temporal bones, derived from micro-CT scans, are employed for surgical training. These models highlight the advantages of tactile interaction, providing a more immersive and practical training experience (26). Such models help trainees appreciate the texture and physicality of anatomical structures, which can be crucial for surgical precision and technique.

Further, research on virtual reality (VR) and augmented reality (AR) systems reveals their potential in enhancing spatial understanding and increasing student engagement. VR and AR technologies offer immersive experiences that can complement traditional learning methods by providing dynamic and interactive visualizations of anatomical structures (27). These technologies allow learners to explore and manipulate 3D models in a virtual environment, which can be particularly beneficial for complex anatomical education.

When compared to traditional 2D systems, our de novo 3D system offers several distinct advantages. Traditional 2D images, such as those found in textbooks or standard medical illustrations, often fail to convey the spatial relationships and depth of anatomical structures effectively. This limitation can hinder a learner's ability to grasp the three-dimensional nature of anatomy, which is essential for understanding spatial orientation and performing clinical procedures.

In contrast, our 3D system provides a comprehensive, three-dimensional perspective of anatomical structures, allowing users to interact with and view models from multiple angles. This capability enhances spatial comprehension and offers a more intuitive understanding of anatomical relationships. Moreover, unlike static 2D images, the dynamic and interactive nature of 3D models facilitates a deeper engagement with the material, supporting more effective learning and retention.

25. Chetan D, Valverde I, Yoo S-J. 3D Printed Models in Cardiology Training. COLLEGE OF CARDIOLOGY FOUNDATION 2024;3.

26. Lähde S, Hirsi Y, Salmi M, Mäkitie A, Sinkkonen ST. Integration of 3D-printed middle ear models and middle ear prostheses in otosurgical training. BMC Medical Education. 2024.

27. Zulfiqar F, Raza R, Khan MO, Arif M, Alvi A, Alam T. Augmented Reality and its Applications in Education: A Systematic Survey. IEEE Access. 2023;11:143250-71.

**Reviewer #2:**

**I really appreciate the effort done in replicating the twin model and the sophistication of**

**methodology listed, but I have a few questions that I couldn't get past regarding the delivered**

**manuscript**

6. Where is the "Korean Standard Full-body Cadaver in metaverse", all what was described was merely bone scans, no flesh, no organs, no full body

Thank you for your valuable comments. As noted, our current research is focused on the initial phase of creating a digital twin from the bone scan of a Korean male cadaver. At this stage, the 3D dataset includes only the bone structures, and does not yet incorporate flesh, organs, or other body parts. Future studies are planned to include these additional anatomical features.

To clarify, the primary goal of this research is to explore complementary approaches for enhancing anatomy education and expanding telemedicine applications. While the initial phase concentrates on the bone data, subsequent research will aim to develop a more comprehensive full-body model.

L100:

Key aspects include the use HoloLens 2 Augmented Reality Head-Mounted Display (AR HMD) technology and real-time cloud rendering for an immersive and interactive educational experience. Initial 3D datasets, comprising extensive anatomical information for each bone, were obtained through the 3D scanning of a full-body cadaver of a Korean male origin. This initial study focuses on the digital twin created from the bone scan of the Korean cadaver, with complementary studies planned to include flesh, organs, and other body parts.

7. You mentioned that this project was assessed by a cohort of medical professionals and students from Ewha Woman’s University and 40 other nursing schools in Korea, where is this assessment.

Thank you for pointing that out.

Since our experiment gathered only open-ended surveys and anonymous feedback from students, professors, and professionals in the medical field, we have revised and specified the part that originally stated “cohort” to “open-ended survey” and “feedback.” Anonymous feedback and quotes have been added to strengthen the validity and clarity of our findings. Accordingly, to ensure anonymity the previously mentioned introduction, “from Ewha Woman’s University and 40 other nursing schools,” has been revised to “from medical professionals and students from nursing schools.”

L136:

Once the 3D DT of the KSMC was successfully imported into the developed Metaverse platform, the pilot test feedback was gathered from anonymous participants in the medical field, including company employees, students and professors from medical and nursing schools. They included open-ended survey from interests, evaluations and adoptability, and suggestions for improvements, which will be found throughout the article. No participants were required to answer all categories but required to answer at least one.

L317:

**Pilot test feedback**

**Group of anonymous college professors**

College professors expressed interest in the applicability of the Metaverse platform across different departments. Majority of their inquiries focused on how the platform could be integrated into school curricula and whether additional content developments would occur to accommodate specific medical departments like ophthalmology, dentistry, cardiology or even veterinary medicine.

**Group of anonymous students**

A participant in an open-ended survey among nursing students who engaged with our program remarked, “Memorizing bone structures from a single diagram has always been a challenge for me. That’s why my friends and I always draw diagrams and color them to memorize them for exams. This metaverse program, however, is very fun interacting with each part of the bone. It’s like floating bones with quiz cards.” Numerous students expressed appreciation for the interactive elements and the capability for real-time collaboration. Another student noted, “When it comes to learning anatomy, I think it’s crucial for us to quiz each other and make the learning process more efficient. However, during the COVID-19 restrictions, it was extremely difficult for us to study because none of us were able to study in groups. If we had been provided with this program, I believe it would have been much easier and more enjoyable to have a group of our classmates join a session together to interact with the same cadaver in real-time.”

8. The name of the study implies the use of metaverse/XR technology to replace traditional teaching with its limitations, why the need for a 3D printing model for bones of the body from the start

Thank you for your insightful comment. We appreciate the opportunity to clarify the rationale behind incorporating 3D printed models in conjunction with Metaverse/XR technology. Accordingly, the contents of the Materials and Methods section were comprehensively revised.

L85:

While the Metaverse/XR technology offers an immersive, interactive experience that allows for detailed exploration and manipulation of anatomical structures, it does not provide the physical feedback and tactile experience that a 3D printed model can offer. The physical 3D printed models allow students and professionals to physically handle and inspect anatomical structures, which can enhance their understanding of spatial relationships and material properties. This is particularly valuable in medical education, where hands-on practice is crucial. Moreover, the 3D DT provides an ethical and readily available alternative to traditional cadaver use, which is often limited by availability and ethical concerns. The physical models can be produced in unlimited quantities, providing consistent, high-quality educational resources that can be accessed without the logistical and ethical issues associated with cadaveric specimens. Thus, the use of both 3D printed models and the Metaverse/XR platform offers a comprehensive educational experience, leveraging the strengths of both physical and virtual modalities to enhance learning outcomes. To provide a comprehensive educational experience that improves learning outcomes by taking advantage of physical and virtual modalities, we 3D printed human bones.

9. What's the importance of 3D printing for the bone scans, despite the fact that plastic models deliver a reasonable accuracy for this particular point that alleviates the need for a 3D print of it.

Thank you for your insightful comments. We recognized that the explanation for this part was insufficient and have provided additional clarification in the paper. 3D printing is not essential for the development of the Metaverse platform itself, but it is a crucial component of the new educational platform we are proposing. The reasons why 3D printing for the bone scans is important in this study are as follows information has been added to the text as per comment number 8.

1. It enables inclusive educational experiences that leverage the strengths of both physical and virtual modalities to improve learning outcomes.
2. The 3D printed model can be used as a display model, allowing pilot test users to experience the difference between a physical 3D model and an immersive 3D digital twin (DT) in the metaverse.
10. Apart of these comments, the rational is acceptable, the methodology is detailed and properly enlisted, but no field studies were added to assess the validity of the proposed tool in anatomical education.

Thank you for your comment. In order to address your comments, we have thoroughly reflected in the materials and methods, results, and discussion sections of the manuscript.

L136:

Once the 3D DT of the KSMC was successfully imported into the developed Metaverse platform, the pilot test feedback was gathered from anonymous participants in the medical field, including company employees, students and professors from medical and nursing schools. They included open-ended survey from interests, evaluations and adoptability, and suggestions for improvements, which will be found throughout the article. No participants were required to answer all categories but required to answer at least one.

L317:

**Pilot test feedback**

**Group of anonymous college professors**

College professors expressed interest in the applicability of the Metaverse platform across different departments. Majority of their inquiries focused on how the platform could be integrated into school curricula and whether additional content developments would occur to accommodate specific medical departments like ophthalmology, dentistry, cardiology or even veterinary medicine.

**Group of anonymous students**

A participant in an open-ended survey among nursing students who engaged with our program remarked, “Memorizing bone structures from a single diagram has always been a challenge for me. That’s why my friends and I always draw diagrams and color them to memorize them for exams. This metaverse program, however, is very fun interacting with each part of the bone. It’s like floating bones with quiz cards.” Numerous students expressed appreciation for the interactive elements and the capability for real-time collaboration. Another student noted, “When it comes to learning anatomy, I think it’s crucial for us to quiz each other and make the learning process more efficient. However, during the COVID-19 restrictions, it was extremely difficult for us to study because none of us were able to study in groups. If we had been provided with this program, I believe it would have been much easier and more enjoyable to have a group of our classmates join a session together to interact with the same cadaver in real-time.”

**Group of anonymous corporate guests**

Although the research and pilot testing were not specific intended for buyers, many corporate guests expressed interest in the pricing of available content on the platform and the overall timeline for the development of the upcoming contents. Meanwhile, some corporate guests were particularly fascinated by the originality of the content and wanted to know if major companies or organizations were already using it. Some reported frequent lags between movements in the scene, which may be due to the quality of internet speed depending on the environment’s connection. This highlights the need for optimize the connection between the platform and the internet to ensure a seamless user experience, regardless of internet speed and stability.

**Feedback from the demo session at a medicine conference**

Feedback from the demo session at an medicine conference highlights the ultimate benefits and necessity of the platform in fields that require an accurate understanding of human anatomy, such as Oriental medicine, particularly acupuncture. Users found that viewing the human skeleton in 3D improved comprehension and teaching performances. They expressed fascination with the interactive features that allowed them to zoom in, zoom out, and rotate the skeletal model. All participants at the demo session were optimistic about this research, envisioning potential collaboration between Western and Asian medicine to form a creative, integrated solution in medical field.

AR elements have been evaluated as effective in improving learners' concentration and immersion. This is a huge advantage in educational settings, promoting better engagement and understanding. The platform's potential to replace traditional hands-on methods using live cadaver dissections or physical models was highly praised for its efficiency. Users highlighted benefits such as cost savings, solving hygiene issues and easing constraints in the physical practice environment.

L406:

The pilot test feedback gathered in this study indicates widespread interest in potential applications across various fields, from education to business within medicine. Key improvements identified include addressing latent challenges related to internet connectivity and lagging, developing tailored content for specific departments, and enhancing platform integration for further business utilization. Additionally, 100% of the pilot test feedback from participants were positive regarding the platform and content’s interactive and immersive features, highlighting its potential to open new doors to traditional learning and training methods in medicine. Moving forward, these valuable insights collected will serve as a steppingstone for further development and optimization of the platform to enhance the complementary research based on the 3D DT of the KSMC. A short video showcases few moments from pilot tests and provides a walkthrough of the developed Metaverse platform and the 3D DT of the KSMC, called ‘Skeletomy.’ This video, included as supplementary material, provides an immersive experience from various perspectives. Two users are featured in the video: the main user (female) is shown from a 1st person perspective, and the secondary user (male) is shown in 3rd person perspective. Together, they demonstrate real-time collaboration features as they interact with the same 3D digital twin of the Korean Standard Male Cadaver. Through this video, viewers also get a chance to immerse themselves in an indirect experience of learning with the KSMC and to envision a new virtual era for applications in anatomy education and telemedicine.

**Manuscript Comments:**

1. Adequate title reflecting content of the paper

Thank you for your comment. The title has been modified to reflect the content of the paper.

L1:

Anatomy Education Potential of the First Digital Twin of a Korean Cadaver

2. Irrelevant

Thank you for your comment. We exclude Login and account creation in Methods and Material section

3. Confusing

Thank you for your comment. We have revised the content to reflect possible confusion to readers.

L273:

The initial screen of the Metaverse platform provides the ability to create rooms where you can interact with other users or join already created rooms. The 'Create Room' feature is launched by clicking the 'Create Room' button in the 'Sharing Session Panel', then follow these steps: First, check if the Provider exists. Once confirmed, we check the number of objects created through the remote rendering service. Once rendered objects are found, remove all objects and run the Provider's room creation function. The Provider's room creation feature checks the names of all rooms created on the currently connected server. If a room with the same name does not exist, create a new room using the room creation feature.

The 'Join Room' feature follows a similar process. When selecting a room to join, the room name set is compared to the existing room. If a matching room is found, the user joins that room. Additionally, when the model location sharing feature is activated, it is integrated with Azure Anchor Service using the configured ID, key, and domain values. This allows remotely rendered models to exist in the same location, with the model's anchor point relative to the person who created the room.

**Existing text**

The acquired ID values are stored in the variables of the SharingServiceProfile script, and when the collaboration feature is initiated, the program automatically connects to the Photon Cloud server using the entered key values. Furthermore, in the Photon Provider configuration, the Provider, an object modularizing overall functionalities used after connecting to the Photon Cloud service, is concurrently configured with the connection to the remote rendering service. The initial launch screen of the metaverse platform offers features such as creating rooms for interaction with others or joining rooms established by other users.

The "Create Room" function, initiated by clicking the 'Create Room' button in the 'Sharing Session Panel,' follows the subsequent process: 1) It first verifies the presence of the Provider. Upon confirmation, it checks the count of objects created through the remote rendering service. 2) Upon identifying rendered objects, it removes all such objects and executes the Provider's room creation function. 3) The room creation function of the Provider examines the names of all rooms created on the currently connected Photon server, and if no room with the same name exists, it utilizes the room creation function provided by the Photon SDK to generate a new room. The "Join Room" function employs the same process, using the same function as the room creation, and when selecting a room to join, it compares the set room name value with existing rooms. If a match is found, the user joins the specified room. Additionally, when the model position sharing feature is enabled, it interfaces with the Azure Anchor Service using the configured Id, Key, and Domain values. This ensures that remotely rendered models coexist in the same location, with the model's reference point based on the person who created the room.
